# Supplementary material for: A UMLS-based spell checker for natural language processing in vaccine safety
Source: BMC Med Inform Decis Mak. 2007 Feb 12;7:3. doi: 10.1186/1472-6947-7-3 (PMC1805499; doi:10.1186/1472-6947-7-3)
Supplement: Additional file 13 — RAP application directory. Sets up directory for RAP (RDF application for PHP) source code [file 1472-6947-7-3-S13.gz › rap/doc/tutorial/usingtheOntModelAPI.htm]

Using RAP's Ontology-Centric OntModel API


### RDF API for PHP V0.9.1

# Using RAP's Ontology-Centric OntModel API

This turorial is part of the RAP - Rdf API for PHP documentation.

Daniel Westphal, Chris Bizer  
October 2004

This tutorial is based on the Jena
Ontology API tutorial*.*

## Table of Contents

1. Introduction
2. Running example: the camera ontology
3. Creating ontology models
4. The generic ontology type: OntResource
5. Handling ontology components: basic class expressions
6. Handling ontology components: properties
7. Instances (individuals)

## Introduction

This tutorial describes RAP's ontology API, and covers a range of common tasks.
Not all details of the API are covered here: Please refer to the RAP PHPDoc to get the full details of
the capabilities of the API.

The ontology model is an extended version of RAP's ResModel API. OntModel extends
ResModel by adding direct support for the kinds of objects expected to be in
an ontology: classes (in a class hierarchy), properties (in a property hierarchy)
and individuals. The properties defined in the ontology language map to accessor
methods. For example, an `OntClass` has a method to list its super-classes,
which corresponds to the values of the `subClassOf` property. This
point is worth emphasizing: no information is stored in the `OntClass`
object itself. When the OntClass `listSuperClasses()` method is called,
the information is retrieved from the underlying RDF model. Similarly adding
a subclass to an `OntClass` asserts an additional RDF statement into
the model.

OntModel just provides a more convenient API for working
with ontology models, but doesn't do any inference by itself. If you also
need inference you have to combine OntModel with an underlying InfModelF or
InfModelB. If you don't need inference you can combine OntModel with an underlying
MemModel or DbModel.

## Running example: the camera ontology

To illustrate the principles of using the Ontology API, we use examples drawn
from a Roger Costello's camera ontology. The camera ontology contains a set
of classes describing some aspects of the domain of still-picture cameras, as
shown below:

We will only use some of the elements from this ontology to
illustrate the ontology API throughout this tutorial.

## Creating Ontology Models

RAP's OntModel is an extension of RAP's ResModel that provides extra
capabilities for handling ontology data sources. Ontology models are created
through the RAP `ModelFactory.`
The simplest way to create an ontology model is as follows:

$ontModel = ModelFactory::getOntModel(MEMMODEL,RDFS\_VOCABULARY);

It uses RDFS as the ontology language and assumes that all of the ontology
data will be stored in-memory (MemModel). Actually only RDFS vocabulary is supported. An OWL vocabulary will be implemented
in the near future

**Note:** it is the choice of reasoner, **not** the choice of language
profile that determines if entailed statements are seen through the ontology
API. The following example creates an ontology model with RDFS entailment in
backward-chaining mode:

$ontModel = ModelFactory::getOntModel(INFMODELB,RDFS\_VOCABULARY);

## The Generic Ontology Type: OntResource

All of the classes in the ontology API that represent ontology values have
`OntResource` as a common super-class. This makes `OntResource`
a good place to put shared functionality for all such classes, and makes a handy
common return value for general methods. The `OntResource` extends
the `ResResource` class, so any general method that accepts a resource
or an `RDFNode` will also
accept an `OntResource`, and consequently, any other ontology value.

Some of the common attributes of ontology resources that are expressed through methods on OntResource are shown below:

| Attribute | Meaning |
| --- | --- |
| comment | A general comment associated with this value |
| label | A human-readable label |
| seeAlso | Another web location to consult for more information about this resource |
| isDefinedBy | A specialization of seeAlso that is intended to supply a definition of this resource |
| sameAs | Denotes another resource that this resource is equivalent to |

For each of these properties, there is a standard pattern of available methods:

| Method | Effect |
| --- | --- |
| add<property> | Add an additional value for the given property |
| set<property> | Remove any existing values for the property, then add the given value |
| list<property> | Return an iterator ranging over the values of the property |
| get<property> | Return the value for the given property, if the resource has one. If not, return null. If it has more than one value, an arbitrary selection is made. |
| has<property> | Return true if there is at least one value for the given property. Depending on the name of the property, this is sometimes is<property> |
| remove<property> | Removes a given value from the values of the property on this resource. Has no effect if the resource does not have that value. |

Example: `addSameAs( $ResResource )`, or `isSameAs( $ResResource )`.

For full details of the individual methods, please consult the PHPDoc.

`OntResource`
defines some other general utility methods. To get or set the value
of a given property, use `addPropertyValue( $property, $value )` or `getPropertyValue( $property )`. Similarly the values of a named property can be listed or removed.

Finally, `OntResource`
provides methods for listing, getting and setting the RDF types of a
resource. The rdf:type property is one for which many entailment rules
are defined in the semantic models of the various ontology languages.
Therefore, the values that `listRDFTypes()` returns is more
than usually dependent on the actual reasoner bound to the ontology
model. For example, suppose we have class A, class B which is a
subclass of A, and resource x whose asserted `rdf:type` is
B. With no reasoner, listing x's RDF types will return only B. If the
reasoner is able to calculate the closure of the subclass hierarchy
(InfModelF and InfmodelB can), X's RDF types would also include A.

For some tasks, getting a complete list of the RDF types of a resource
is exactly what is needed. For other tasks, this is not the case. An
ontology editor, for example, might want to distinguish in its display
between inferred and asserted types. In the above example, only `x rdf:type B`
is asserted, everything else is inferred. One way to make this
distinction is to make use of the base model. Getting
the x resource from the base model and listing the type properties
there would return only the asserted values.

To list the RDF types of a resource, use:

listRDFTypes() // assumes not-direct   
listRDFTypes( true ) // if true, show only direct relationships

Related methods allow the `rdf:type` to be tested, set and returned.

## Handling Ontology Components: Basic Class Expressions

Classes are the basic building blocks of an ontology. A simple class is represented
in RAP by an OntClass
object. You can get an OntClass by calling `getOntClass()` on the ontology
model:

$camera = $ontModel->createOntClass( "camNS"."Camera" );

Once we have the ontology class object, we can begin processing it through
the methods defined on `OntClass`. The attributes of a class are
handled in a similar way to the attributes of OntResource, above, with a collection
of methods to set, add, get, test, list and remove values. Class properties
that are handled in this way are:

| Attribute | Meaning |
| --- | --- |
| subClass | A subclass of this class, i.e. those classes that are declared `subClassOf` this class. |
| superClass | A super-class of this class, i.e. a class that this class is a `subClassOf`. |

Thus, in our example ontology, we can print a list the subclasses of Camera as follows:

$camera = $model->createOntClass( "camNS:" . "Camera" );   
foreach ($camera->listSubClasses() as $resResource)   
{  
   echo $resResource->getLabel();  
}

OntClass has some other commonly used utilities. To show all of the instances that mention this class as their `rdf:type` (or one of them), use `listInstances()`.

## Handling Ontology Components: Properties

OntProperty is an extension of `OntResource` and allows access to the additional information
that can be asserted about properties in an ontology language Again, using the
pattern of add, set, get, list, has, and remove methods, we can access the following
attributes of an `OntProperty`:

| Attribute | Meaning |
| --- | --- |
| subProperty | A sub property of this property; i.e. a property which is declared to be a `subPropertyOf` this property. If p is a sub property of q, and we know that `A p B` is true, we can infer that `A q B` is also true. |
| superProperty | A super property of this property, i.e. a property that this property is a `subPropertyOf` |
| domain | Denotes the class or classes that form the domain of this property. Multiple domain values are interpreted as a conjunction. The domain denotes the class of value the property maps from. |
| range | Denotes the class or classes that form the range of this property. Multiple range values are interpreted as a conjunction. The range denotes the class of values the property maps to. |

In the example camera ontology, the property body is a sub-property of
part, and has domain Camera and range Body (that is, it maps from
instances of cameras to instances of camera bodies). We can reconstruct
this definition in an empty ontology model as follows:

$newM = ModelFactory::getOntModel(MEMMODEL,RDFS\_VOCABULARY);   
$camera = $newM->createOntClass( "camNS:". "Camera" );   
$body = $newM->createOntClass("camNS:" . "Body" );   
  
$part = $newM->createOntProperty("camNS:". "part" );   
$body = $newM->createOntProperty("camNS:". "body" );   
  
$body->addSuperProperty( $part );   
$body->addDomain( $camera ); $body->addRange( $body );

## Instances (Individuals)

RAP supports a simple notion of an `Individual,` which is essentially
an alias for ResResource.

$camera = $newM->createOntClass( "camNS:". "Camera" );   
$instance = $camera->createInstance("camNS:". "CANJI JXP3");

In the above example, the individual is named, but this is not necessary.
The method call `createInstance()` creates an anonymous individual
belonging to the given class.
